# Emergent behavioural phenotypes of swarming models revealed by mimicking a frustrated anti-ferromagnet

**DOI:** 10.1098/rsif.2015.0520

**Published:** 2015-10-06

**Authors:** D. J. G. Pearce, M. S. Turner

**Affiliations:** 1 Department of Physics, University of Warwick, Coventry CV4 7AL, UK; 2 MOAC Doctoral Training Centre, University of Warwick, Coventry CV4 7AL, UK; 3 Centre for Complexity Science, University of Warwick, Coventry CV4 7AL, UK

**Keywords:** swarm, animal behaviour, collective motion, active matter, self-propelled particles, frustration

## Abstract

Self-propelled particle (SPP) models are often compared with animal swarms. However, the collective animal behaviour observed in experiments often leaves considerable unconstrained freedom in the structure of a proposed model. Essentially, multiple models can describe the observed behaviour of animal swarms in simple environments. To tackle this degeneracy, we study swarms of SPPs in non-trivial environments as a new approach to distinguish between candidate models. We restrict swarms of SPPs to circular (periodic) channels where they polarize in one of two directions (like spins) and permit information to pass through windows between neighbouring channels. Co-alignment between particles then couples the channels (anti-ferromagnetically) so that they tend to counter-rotate. We study channels arranged to mimic a geometrically frustrated anti-ferromagnet and show how the effects of this frustration allow us to better distinguish between SPP models. Similar experiments could therefore improve our understanding of collective motion in animals. Finally, we discuss how the spin analogy can be exploited to construct universal logic gates, and therefore swarming systems that can function as Turing machines.

## 1. Background

Collective motion in large groups of animals represents one of the most conspicuous displays of emergent order in nature [1–4]. The idea that such swarms manifest some kind of effective group intelligence has been explored in several recent studies [5–8]. While swarming is ubiquitous in nature, it is still surprisingly poorly understood. In particular, there is a large space of candidate agent-based models, some of which have been studied in detail [9–20]. Typically, a rule for the motion of every individual is first specified and the resulting collective motion is then studied. However, it can be very difficult to refine this ‘microscopic’ rule by studying data for the collective ‘macroscopic’ behaviour. By observing swarms of a limited size, it is possible to see what reaction an individual has to its immediate neighbours and infer a set of rules that give rise to the observed behaviour [21–23]. While informative, this approach still leaves structural freedom in how one constructs a model to give rise to the observed behaviour. Another method is to take a maximum entropy approach [24–26], finding the model with the minimum structure that is consistent with observations. This technique has been used to show that pairwise interactions are sufficient to explain order propagation through the entirety of a flock of starlings and support the conclusion that interactions governing starling flocks are topological in nature [24]. Despite these methods, the essential difficulty of model building still remains: it is an inverse problem in which no complete set of techniques yet exist to perform this inversion.

Recent experiments have used confined environments that restrict motion to further probe the underlying behaviour of animal swarms [27–31] even managing to create behaviour that mimics logical operations [32,33]. Our work was primarily motivated by one such experiment performed on locusts enclosed in a single ring-shaped channel [27] where increasing the density of locusts results in a transition from a state of random motion to a polarized state in which the locusts co-align to create coherent, circulating swarms. Due to the ring-shaped enclosure, the swarm was able to polarize into clockwise or anticlockwise circulation, giving it a spin-like nature. This same approach has since been applied to study fish shoals [29].

**Figure 1.**
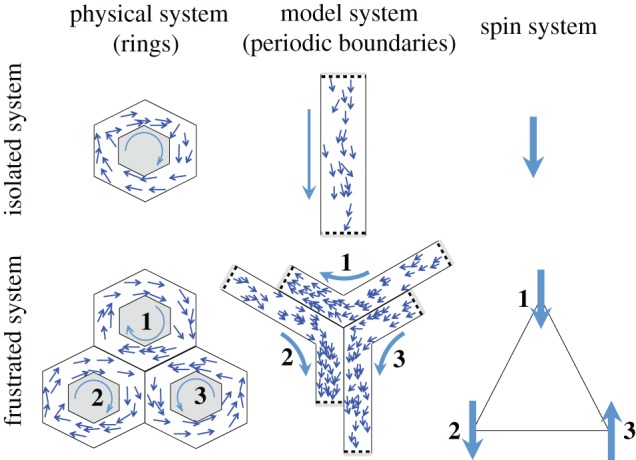
Different SPP models are studied in confining channels. *Isolated system*: the macroscopic behaviour of a ring containing swarming animals is approximated by interacting agents moving in a linear, semi-periodic channel, for simplicity. Clockwise/anticlockwise collective motion in the ring, analogous to a spin, corresponds to motion up/down the semi-periodic channel. *Frustrated system*: the motion within three rings arranged on a triangular lattice is frustrated when interactions are permitted across windows between the tracks. This is again simulated using linear semi-periodic channels for the SPP model (which remain linear but are shown as kinked in the middle panel for clarity; periodic linear channels with windows between all pairs cannot easily be represented in a two-dimensional image). This system is analogous to a geometrically frustrated anti-ferromagnet.

This behaviour was compared with a simple one-dimensional self-propelled particle (SPP) model with periodic boundary conditions (see the *isolated system* panels in figure 1). The polarization transition and the mean time between spontaneous polarization inversions were then related to the parameters of the model [27]. However, we believe that it is hard to draw any definite conclusions concerning the correct *structure* for the model as there remains considerable freedom to choose structurally and parametrically distinct SPP models that would all be capable of reproducing this stylized behaviour. It is a challenging task to distinguish structurally distinct candidate models by comparison with data like this. Our approach is to seek to break the behavioural degeneracy between models, in particular to reveal new macroscopic behaviours specific to the microscopic rules of interaction. In order to achieve this, we first consider two ring-shaped channels arranged near to one another that share a (section of) boundary through which the individuals can pass information but cannot physically cross. This could be realized experimentally by connecting the rings by a window. In animals that mainly employ a sense of vision, a transparent window or the use of images recorded from one ring and projected onto another might be appropriate; for animals that use touch a limited physical opening might be used. Interactions between swarms on either side of a glass window [31] and with projected images [34,35] have been observed experimentally. For active particles that interact by hydrodynamic or electromagnetic effect, physical proximity can be used to observe an interaction between distinct swarms [36,37]. This window provides a coupling between the two rings. Here, we extend the interactions between individuals to include neighbours that are *visible* through the window, as well as those that are visible within the same ring, and use the same behavioural rule for both cases. For highly polarized swarms, driven by co-alignment, we would then expect a ring polarized anticlockwise (an ‘up’ spin) to be most stable when it is adjacent to a ring polarized clockwise (a ‘down’ spin), or vice versa: only in this situation would neighbours connected through the window also find themselves co-aligned. The coupling across the window is therefore anti-ferromagnetic in character.

Inspired by the extensive literature on frustrated anti-ferromagnetic systems [38–40], we analyse motion in three rings arranged so they each share a boundary with the other two (see the *frustrated system* panels in figure 1). In this way, we create a system similar to geometrically frustrated anti-ferromagnetic atoms on a triangular lattice. It is no longer possible for all three rings to remain highly polarized and co-aligned across all windows. As in the analogous magnetic system, we no longer expect a unique pair of symmetric ground states to exist. We anticipate that additional information can be obtained from the resulting behaviour, whatever it may be, that can be used to better distinguish microscopic models when they are constrained against observed behaviour.

## 2. Material and methods

In what follows, we compare two different SPP models frustrated in this way. Apart from the boundary conditions, both take a fairly standard form in which *N* particles move in a periodic box with a constant speed *v*_0_ = 1. When combined with a (unit) time step, this defines our units of length throughout. At each discrete time step, every particle orientates its velocity along the average direction of motion of its neighbours. The only difference between the two models studied here will be how these neighbours are identified. Writing those neighbours to the *i*th particle as 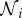, the equation of motion involves the average velocity of its neighbours 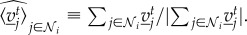 Noise is introduced by randomly orientated unit vectors 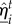 that are uncorrelated between individuals and in time 
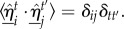
 The position, 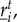 and velocity, 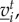 of particle *i* at time *t* are then given by the following equations, where the parameter *ϕ*_n_ < 1 controls the relative weighting of the noise term and a hat 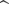 indicates a unit vector throughout:2.1
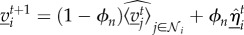
and2.2
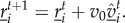


The first of our models is typical of a class that identify nearest neighbours according to a *metric*-based measure of distance (the model due to Vicsek *et al*. [10] is often cited as a prototype). Here, a particle co-aligns with others that lie within a fixed interaction range *R*. This definition means that individuals can have as few as zero or as many as *N* − 1 neighbours. The second model selects nearest neighbours according to a *metric-free* scheme, motivated by the evidence for interactions with this character in bird flocks [41,42]. In this model, each particle aligns with the *N_c_* nearest particles, irrespective of absolute separation. While other choices of candidate model are possible, most obviously spatially balanced metric-free models in which individuals interact with neighbours identified by Voronoi tessellation [13,43] or their relative angular position [44], we restrict our study to the two selected as they are very similar in structure and both have a tuneable interaction range.

Both of the candidate models generally exhibit two distinct states: *ordered*, in which the particles achieve a high level of polarization and all their velocities are locally highly aligned, and *disordered*, in which there is no net polarization and the velocities of individuals are largely uncorrelated. The transition from the disordered to ordered state is primarily controlled by two quantities: the noise weighting, *ϕ*_n_, and the density of particles. For sufficiently low noise and high density, the system is ordered. As the noise is increased (or the density is decreased), the system undergoes a transition into the disordered state. Here, we simulate swarms of *N* = 100 SPPs in a semi-periodic box of width and height *W* = *H* = 2.5 and length *L* = 25 in the *x*-, *y*- and *z*-direction, respectively. This is an unconventional choice in that the system is only periodic in the *z*-direction, instead of in *x*, *y* and *z*. If a particle reaches a boundary perpendicular to the *x*- or *y*-directions, it undergoes an elastic collision, or reflection, in which the component of its velocity perpendicular to that boundary is reversed. In this way, the swarm can be confined to a slender, periodic channel (see the electronic supplementary material for details). This leaves three free control parameters, the number of particles, *N*, the noise weighting, *ϕ*_n_, and the interaction range, *R*, for the metric and *N_c_* for the metric-free models (see the electronic supplementary material for details). Simulations were computed for 90 000 time steps after a 10 000 time step pre-equilibration (except for the spatial inhomogeneity data where 10 realizations each of 10 000 time steps were more appropriate).

Due to the nature of the semi-periodic box, the swarm cannot sustain a high level of polarization unless it is aligned nearly parallel, or anti-parallel, to the *z*-axis. This is because orientation in either the *x*- or *y*-direction will result in collisions with the non-periodic boundaries and the individuals in the swarm will then rapidly change direction in an incoherent fashion until order along *z* re-emerges. For this reason, it is possible to quantify the polarization of the system using only the *z*-component of velocity, analogous to the polarization of circulation:2.3
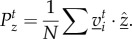


For disordered swarms 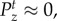 and for highly ordered swarms 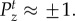


**Figure 2.**
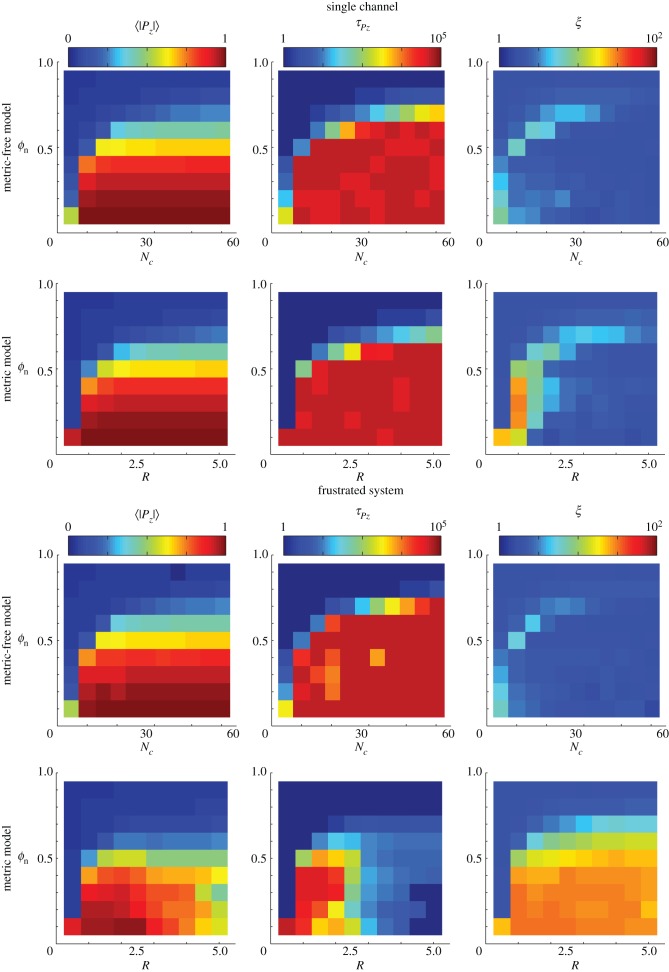
The behaviour of the metric and metric-free models can be more easily distinguished when the system is frustrated. Simulations are performed in a single channel (top six panels) and a system of three fully frustrated channels (bottom six panels). Shown is the average polarization magnitude (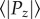, left column), polarization correlation, or persistence, time (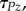 middle column), and the spatial inhomogeneity (*ξ*, right column) for various interaction ranges (*R* or *N_c_* for metric or metric-free models, respectively) and noise levels (*ϕ*_n_). See text for details. The persistence time and spatial inhomogeneity are represented on logarithmic scales.

Swarms of SPPs confined in these channels support both ordered and disordered phases (with high and low polarizations, respectively), with a transition between the two around *ϕ*_n_ ∼ 0.5 (see figure 2 (single channel)). Near this transition, the swarms are polarized, 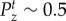, and have a clear direction of motion along the channel, but there is still sufficient noise that the swarm can reverse direction, evidenced by the autocorrelation times for 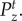 As *ϕ*_n_ is decreased, the rate of these directional switches decreases and the direction of polarization eventually no longer changes on time scales that are accessible in our simulations. A similar outcome is observed for both SPP models, reproducing the behaviour of insect swarms enclosed in a ring and previous simulations thereof [27].

**Figure 3.**
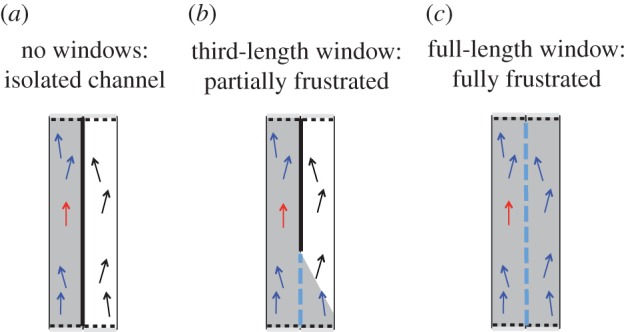
A sketch showing how the length of the windows can be varied to adjust the degree to which the channels are frustrated. (*a*) No windows therefore isolated channels with no frustration, (*b*) third-length windows, hence a partially frustrated system, and (*c*) full-length windows give a fully frustrated system. In all cases, the red individual is able to interact with any of the (blue) individuals to which there is an unbroken (by a boundary) line of sight, i.e. within the grey areas.

In order to introduce a coupling between two adjacent channels, they are positioned alongside each other so that they share a face normal to the *y*-axis (say), i.e. particles in channel 1 can be thought of as being restricted to 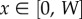 and particles in channel 2 to 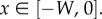 This means that the minimum distance between two particles in different channels is zero and particles in different channels can co-align if the line-of-sight connecting them passes through a region designated as a window. No transport of particles is allowed across the window. We can adjust the degree of coupling between two channels by changing the length of the windows (figure 3). Since we are not restricted by geometrical considerations in these simulations, it is possible to extend the windows to run along the full length of the channel, with each channel sharing such a window with each of the other channels. We refer to this as a fully frustrated system. With pairwise coupling between three channels, we can arrange them so as to be mutually frustrating (figure 1). We restrict our study, and hence conclusions, to finite systems. Although we are employing periodic boundaries in our simulations, this is not done to recreate an infinite area, rather as an analogue to a circular track. Further to this, the infinite size limit becomes hard to define when partial frustration is introduced, as the windows do not span the entire length of the channel; they require the system to be periodic rather than infinite.

## 3. Discussion

**Figure 4.**
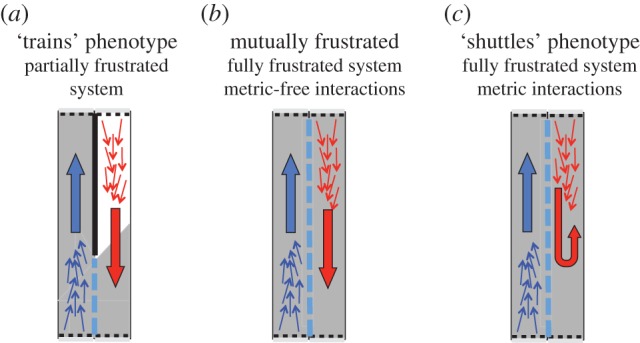
Sketch showing the emergent phenotypes from swarms simulated in frustrated channels. (*a*) The ‘trains’ phenotype involves swarms avoiding each other by passing the windows at different times; this is only possible in the partially frustrated system (see electronic supplementary material, movie S1). (*b*) In the mutually frustrated phenotype swarms pass by each other with minimal interaction; this is only observed for the metric-free swarms (see electronic supplementary material, movies S2 and S4). (*c*) When the interaction range is large metric, swarms cannot pass by each other without interacting and one swarm reversing its direction. This leads to reduced persistence times (see electronic supplementary material, movie S3).

The results of simulations of such fully frustrated systems are as follows (see the electronic supplementary material for details). For high noise, or very low interaction range, both swarms occupy a disordered state. If the noise is sufficiently low, and the interaction range sufficiently high, both swarms are able to adopt a highly polarized state. For weak interactions (short range *R* or small number *N_c_*), little difference is observed in the behaviour of the swarms, both having a polarized state which rarely changes direction (figure 2 (frustrated system)). As the interaction range is further increased, both swarms remain highly polarized but SPPs with metric interactions show a sudden reduction in the directional switching time, 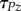, around *R* ≈ 2.5. This is because when the metric interaction range becomes comparable to the width of the channels, *W* ≈ *R*, two swarms in adjacent channels are unable to pass by each other without interacting. This often results in one of them reversing direction, a behaviour similar to ‘shuttles’ going back and forth (figure 4*c*). It also acts to push the swarms into high-density bands since the leading front is the first to be affected by a band in another channel (see electronic supplementary material, movies). This is evidenced by the higher values of *ξ*, defined as the maximum time-averaged variance in the number of particles observed in any constant fraction of the channel length. In contrast to this, SPPs with metric-free interactions exhibit high polarization and long polarization autocorrelation times, 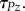 As these swarms clump into bands, the majority of nearest neighbours remain sited in the same channel, which leads to a weaker coupling between swarms in adjacent channels; this allows them to pass each other without a significant effect on the polarization. Hence the fall in persistence times is not seen for metric-free swarms; we call this state ‘mutually frustrated’ (figure 4*b*). We also studied partially frustrated systems in which the windows extend over only a third of their length (figure 3*b*; this resembles the physical system sketched in the bottom left panel of figure 1). For low interaction range, the partially frustrated system shows qualitatively similar effects to the fully frustrated system (see the electronic supplementary material). When the interaction range is increased, the metric swarm adopts a phenotype in which the swarms in adjacent channels each pass the window at different times, hence the other swarm is always in another part of the ring. We refer to this as the ‘trains’ behavioural phenotype (see figure 4*a* and electronic supplementary material, movies).

## 4. Creating an information processing device

Swarms, both simulated and observed, are often unpredictable and stochastic in their nature. While we may be able to use statistical methods to predict how certain macroscopic values may vary, such as the polarization, the exact behaviour of a swarm is often entirely random. For example, we know the location and nature of the order transition in the Vicsek model, but the direction in which the swarm polarizes when the symmetry is broken is entirely unpredictable. When the swarm is confined to an elongated channel, as is the case here, the possible directions in which the swarm can polarize is now reduced to two, equally likely, outcomes. In the final part of this paper, we explore further the spin-like nature of the motion within these channels to make the swarm predictable and influenceable. This can be achieved by employing a specific channel geometry to construct an information processing device.

**Figure 5.**
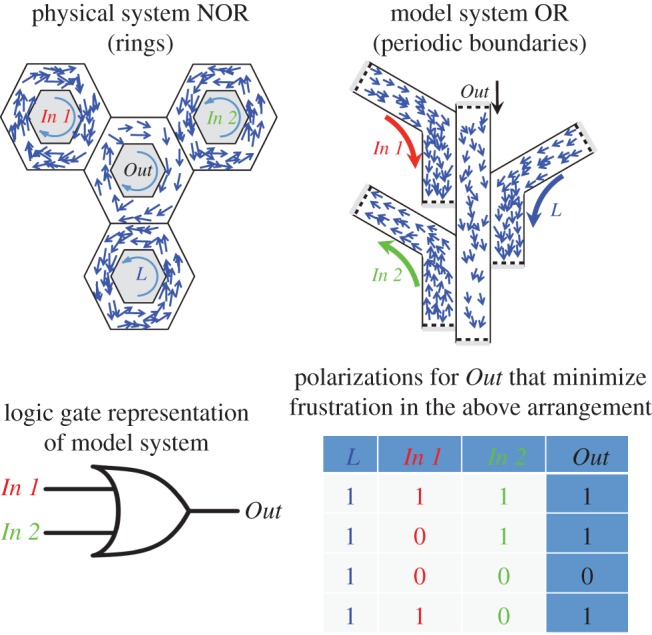
Arrangements of interacting rings (physical system) or periodic channels (model system) containing SPPs, as a model for animal systems, that are predicted to perform logical operations by minimizing the overall frustration in the system, with the direction of polarization of the *Out* ring the output, and *In 1* and *In 2* the inputs. We study a model SPP system in the corresponding arrangement of periodic channels. The table shows the polarization directions for the *Out* channel (right column) that minimize frustration for different combinations of input polarizations (middle columns); this corresponds to the output of a logical OR gate.

We consider the arrangements of channels shown in figure 5. Here, the polarization of the *Out* channel depends on the polarizations of channels *In 1*, *In 2* and *L* (for Locked). In the arrangement shown in figure 5 (physical system), if one takes clockwise and anticlockwise as 0 and 1, respectively, adopting a state that minimizes the overall frustration would lead to a logical NOR (for *L* locked anticlockwise) or NAND (for *L* locked clockwise) type response (an OR or AND response could also be achieved by placing the *In 1*, *In 2* and *L* rings inside the *Out* ring). We apply a similar approach to the arrangement shown in figure 5 (model system) and using a ‘bit’ defined as4.1
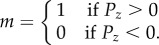

The choice of the positive *z*-direction is somewhat arbitrary between channels; here we employ the convention that aligned swarms in adjacent channels will have *P_z_* of the same sign. This arrangement would (and does) lead to the logic table shown in figure 5, which is equivalent to an OR gate.

**Figure 6.**
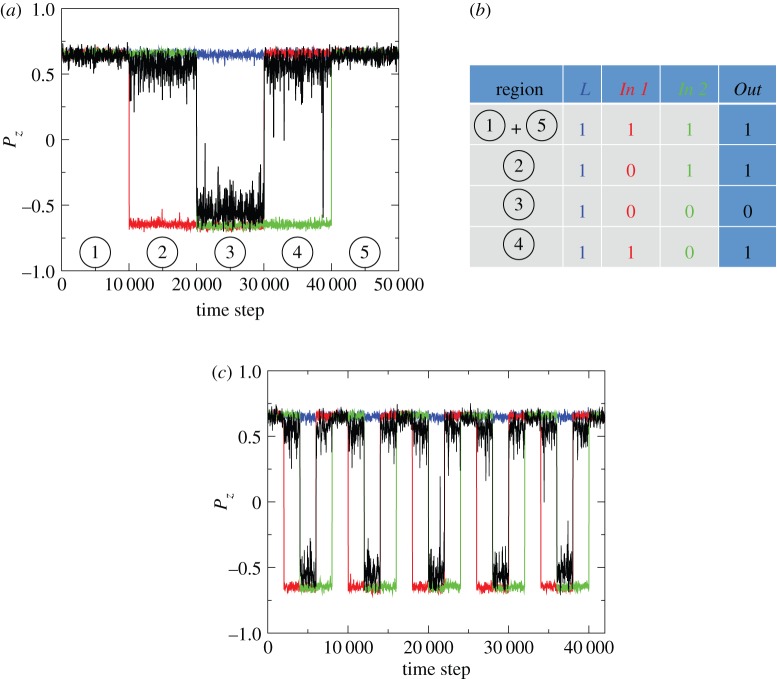
The polarization of SPPs with metric-based interactions moving in the system shown in figure 5. The polarization in each channel is recorded over the course of a simulation run in which the particle polarizations in the *In 1* (red trace) and *In 2* (green trace) channels are manually inverted at intervals of (*a*) 10 000 and (*c*) 2000 time steps. Also shown is the *Out* channel (black trace) and Locked channel (designated *L*, blue trace), the latter maintaining a polarization in the +*z*-direction throughout. After inversion, the polarization of the *Out* channel undergoes a spontaneous and rapid transition to the state shown in the table in (*b*). Such systems can therefore mimic the behaviour of a deterministic logic gate. Electronic supplementary material, movie S5, shows a simulation of logical NOR cycling through inputs in a similar fashion.

To validate that the logic table shown in figure 5 is indeed realized, we employ SPPs with metric-based interactions of range *R* = 2.5 and noise level *ϕ*_n_ = 0.5. In the absence of frustration, this would lead to moderately polarized swarms with long persistence times (figure 2). We include fewer particles in the *Out* channel, making it more likely to switch when it interacts with a larger swarm. We also assign it a width that is smaller than the interaction radius; this ensures it is strongly influenced by adjacent swarms effectively reducing the distance between them. These differences mean that the *Out* channel is more likely to rapidly reverse direction than the *In* and *L* channels when it is frustrated (see the electronic supplementary material for details). This results in an essentially deterministic logical output, rather than one that is only realized statistically, because the *Out* channel responds to the *In* channels, and not the other way around; effectively we do not expect the *In* and *L* channels to spontaneously reverse polarization. In order to probe the system, we manually invert the directions of all particles in either of the *In* channels and observe the response of the swarm in the *Out* channel. When the swarms in the *In* channels are periodically switched over a full range of inputs for a logic gate, the *Out* channel is seen to respond in a way that is consistent with the operation of a logical OR (figure 6*a*). Figure 6*c* shows that the system continues to recreate the response of a logical OR over multiple cycles, even when the switching rate is increased. In both these cases, the *Out* channel gives the correct response for over 99% of the simulation. We can also replicate the ring geometry shown in figure 5 (physical system) to achieve a logical NOR response with respect to clockwise/anticlockwise polarization (see electronic supplementary material, movie S5).

## 5. Conclusion

In summary, we show that different models can better be distinguished when the particle (animal) motion is frustrated. We achieved this by introducing windows through which particles confined to different channels can interact. We then use a channel geometry that mimics a geometrically frustrated anti-ferromagnet. This approach can be applied to any model for collective motion in which particles interact; with the correct engineering of a suitable window, it may be possible to apply this to experimental systems. This method promises to allow us to better distinguish between models for animal behaviour by comparing them with experimental data that is itself obtained in frustrated geometries. Ultimately, this could lead to an improved insight into the behavioural mechanisms that lead to swarming, one of the prototypical examples of emergent order in nature.

Finally, we use a spin analogy to propose confining geometries in which the swarm(s) perform the operation of a universal logic gate. The behaviour of a swarm is inherently stochastic. By applying certain geometrical constraints, we have managed to make certain aspects of a swarm's behaviour predictable, and even influence them, here recreating the operation of a logic gate. These could be combined to perform more complex computational tasks, placing a bound on the computational capability of animal swarms, at least those that are artificially confined in this way, to that of a Turing machine.
